# Trichostatin A Affects Developmental Reprogramming of Bread Wheat Microspores towards an Embryogenic Route

**DOI:** 10.3390/plants9111442

**Published:** 2020-10-26

**Authors:** Ana María Castillo, Isabel Valero-Rubira, María Ángela Burrell, Sandra Allué, María Asunción Costar, María Pilar Vallés

**Affiliations:** 1 Departamento de Genética y Producción Vegetal, Estación Experimental de Aula Dei, Consejo Superior de Investigaciones Científicas (EEAD-CSIC), Avda Montañana 1005, 50059 Zaragoza, Spain; amcast@eead.csic.es (A.M.C.); ivalero@eead.csic.es (I.V.-R.); sallue@eead.csic.es (S.A.); acostar@eead.csic.es (M.A.C.); 2 Departamento de Patología, Anatomía y Fisiología, Facultad de Ciencias, Universidad de Navarra, C/Irrunlarrea s/n, 31008 Pamplona, Spain; mburrell@unav.es

**Keywords:** bread wheat, microspore embryogenesis (ME), trichostatin A (TSA), doubled haploid (DH), ultrastructural characterization, expression of ME marker genes

## Abstract

Microspores can be developmentally reprogrammed by the application of different stress treatments to initiate an embryogenic pathway leading to the production of doubled haploid (DH) plants. Epigenetic modifications are involved in cell reprogramming and totipotency in response to stress. To increase microspore embryogenesis (ME) efficiency in bread wheat, the effect of the histone deacetylase inhibitor trichostatin A (TSA) has been examined in two cultivars of wheat with different microspore embryogenesis response. Diverse strategies were assayed using 0–0.4 µM TSA as a single induction treatment and after or simultaneously with cold or mannitol stresses. The highest efficiency was achieved when 0.4 µM TSA was applied to anthers for 5 days simultaneously with a 0.7 M mannitol treatment, producing a four times greater number of green DH plants than mannitol. Ultrastructural studies by transmission electron microscopy indicated that mannitol with TSA and mannitol treatments induced similar morphological changes in early stages of microspore reprogramming, although TSA increased the number of microspores with ’star-like’ morphology and symmetric divisions. The effect of TSA on the transcript level of four ME marker genes indicated that the early signaling pathways in ME, involving the *TaTDP1* and *TAA1b* genes, may be mediated by changes in acetylation patterns of histones and/or other proteins.

## 1. Introduction

In microspore embryogenesis (ME), the fate of the microspore towards the development of a pollen grain is diverted to follow an embryogenic pathway. This developmental change is generally triggered in response to a stress treatment, followed by an in vitro culture phase leading to the formation of multicellular structures that differentiate into embryos and finally in haploid plants [1]. Doubled haploids plants (DH) can be obtained spontaneously by nuclear fusion or endoreduplication or can be induced by the application of chromosome doubling agents [2,3]. The value of DH plants for the production of new varieties or inbred lines is undoubted since complete homozygosis is obtained in one generation. Furthermore, phenotypic selection efficiency performed on DH population is substantially increased compared to conventional methods. Therefore, breeders can release a new variety or inbred line in 4–6 years. Moreover, the use of DH plants in combination with marker-assisted selection or gene editing techniques presents a considerable potential to shorten the breeding cycles [4].

The production of DH lines is particularly relevant in species such as wheat (*Triticum aestivum* L.), since there is a great demand for varieties adapted to new climatic conditions to achieve the objective of increasing wheat production by 38% by 2050 [5]. However, bread wheat ME protocols developed until now have a strong genotype-dependence, with low frequencies of green DH plants obtained in many genotypes [6,7], mainly due to the limited number of microspores that are able to successfully change the pattern of development [8].

Morphological changes characterize the developmental transition from the microspore to an embryogenic structure. The movement of the nucleus to a central position together with a fragmented vacuole by cytoplasmic strands (‘star-like’ morphology, SLM), promoting an initial symmetric division, should be highlighted [1]. These modifications are the result of altered gene expression, by the silencing of genes involved in the gametophytic program or by activation of genes of the embryogenic pathway. These changes are accompanied by the expression of genes related to stress response, chromatin modification, hormonal signal transduction, and secondary metabolism [9,10,11,12].

Transcription is precisely regulated by modifications of chromatin structure to ensure gene expression adjustment to cell differentiation and development [13,14,15]. Changes in DNA methylation and post-translational histone modifications, mainly methylation and acetylation, have been associated with ME induction in model species such as *Brassica napus* and *Hordeum vulgare* (for a review, see [16]). A global DNA hypomethylation, a low level of histone H3K9 methylation (H3K9me2), and a high level of H3 and H4 acetylation (H3Ac and H4Ac) have been correlated with totipotency in microspore reprogramming, whereas de novo DNA methylation, a high level of H3 methylation and a marked decrease in histone acetylation have been described at the proembryo stage (for a review, see [16]).

In recent years, the acquisition of knowledge about the epigenetic mechanisms involved in microspore reprogramming has allowed the development of new strategies to improve the efficiency of ME induction. Strategies based on the application of inhibitors of enzymes involved in modification of epigenetic marks have been successfully addressed in the model ME species *Brassica* and barley. Thus, the enhancement of DNA hypomethylation by treatment with 5-azacitydine (AzaC), an inhibitor of DNA methyl transferase activity, successfully increased the induction of ME [17]. The same effect was described in triticale by the application of AzaC during the last days of a cold treatment [18]. In addition, modifying histone methylation by the histone lysine methyltransferase G9a inhibitor, BIX-01294, also raised embryogenesis induction [19].

Finally, the application of histone deacetylase inhibitors, such as trichostatin A (TSA), sodium butyrate (NaB) and vorinostat (SAHA), that block the removal of acetyl groups from Lys residues, also increased the efficiency of microspore reprogramming in *Brassica* and barley [20,21,22]. In wheat, the application of TSA to isolated microspores from cold-treated spikes during the isolation procedure or the culture phase increased the efficiency of DH production [23,24]. However, the optimal concentration of the inhibitor, the application time, and its effect in combination with stress treatments used in anther or microspore cultures, have not been investigated in detail.

For the first time, a comprehensive comparison of TSA application in combination with or without standard stress treatments has been performed in wheat anther culture to induce ME. Key variables for the production of breeding DH populations, such as the numbers of green plants and green DH plants, and the percentages of green plants and spontaneous chromosome doubling have been studied. The effect of DMSO as solvent for TSA was also analyzed. The greatest number of green DH plants was obtained with the application of TSA simultaneously with mannitol treatment. Morphological and ultrastructural characterization of microspore reprogramming after inductive treatments indicated that the application of TSA with mannitol trigger similar morphological changes to those of mannitol. However, TSA with mannitol increased the number of embryogenic microspores. TSA-induced changes in the levels of expression of marker genes for early stages of wheat ME suggested that an increase in histone acetylation and/or other proteins regulated the signaling pathways in early ME.

## 2. Results

### 2.1. Application of a Short TSA Treatment after a Cold or Mannitol Stress Treatment

Different concentrations of TSA (0, 0.1, 0.2, and 0.4 µM) were applied for a short time (2 h) to anthers from cold-treated spikes for 7–9 days at 4 °C (CLD) (Supplementary Figure S1A) or 0.7 M mannitol-treated anthers for 5 days at 25 °C (MAN) (Supplementary Figure S1B) from high responding cv. Pavon and medium-low responding cv. Caramba. ANOVA showed that independent of the type of stress treatment applied, significant differences between the two cultivars were found for all variables. As expected, Pavon produced higher values than Caramba (data not shown). It should be noted that control cultures after MAN produced a 4 times greater number of proembryos, 2 times the number of embryos, almost 6 times the number of green plants, and a 3.5 times higher percentage of green plants than after CLD (Table 1 options A, B). However, the percentage of spontaneous chromosome doubling in CLD was 3 times higher than in MAN.

When TSA was applied after a 7–9-days cold treatment (C+0T, C+0.1T, C+0.2T, and C+0.4T), neither significant differences among treatments nor with control cultures (CLD) were found for any of the variables studied (Table 1 option A). Only C+0.2T and C+0.4T had a tendency to reduce the percentage of green plants and increase the percentage of spontaneous chromosome doubling in comparison to CLD and C+0T (cold plus 1% DMSO). When TSA was applied after a 5-day 0.7 M mannitol treatment at 25 °C (M+0T, M+0.1T, M+0.2T, and M+0.4T), no significant differences among treatments were observed for any of the variables except for the percentage of spontaneous doubling (Table 1 option B). The highest percentage was reached with M+0.1T, rendering a 2- and only a 0.5-fold increase compared with M+0T (mannitol plus 1% DMSO) and MAN, respectively. Interestingly, DMSO (M+0T) had a tendency to reduce the variable number of green plants, but M+0.1T produced similar values to MAN. The combination of both variables resulted in a 3- and 1.6 times higher number of green DH plants in M+0.1T (4.3) than in M+0T (1.4) and MAN (2.7).

### 2.2. Application of TSA Simultaneously with a Cold or a Mannitol Stress Treatment 

The effectiveness of the application of TSA at 0, 0.1, 0.2, and 0.4 µM simultaneously with CLD and MAN was assayed (Supplementary Figure S1C,D, respectively). ANOVA showed that concentration of 0.2 and 0.4 µM TSA with CLD (C0.2T and C0.4T, respectively) significantly increased the numbers of proembryos, embryos, and green plants compared with the controls C0T (cold with 0.1% DMSO) and CLD (Table 1 option C). The C0.4T treatment was the most efficient, rendering up to a 1.7-fold increase in the number of proembryos and embryos and a 3.2-fold increase in the number of green plants with respect to CLD. In contrast, the percentage of spontaneous doubling decreased from 81.6 in CLD or 76.8 in C0T to 63.5 in C0.4T. Consequently, C0.4T resulted in a 2.5 to a 3.5 times higher number of green DH plants than CLD and C0T (3.3, 1.3, and 0.9 with C0.4T, CLD, and C0T, respectively).

**Figure 1:**
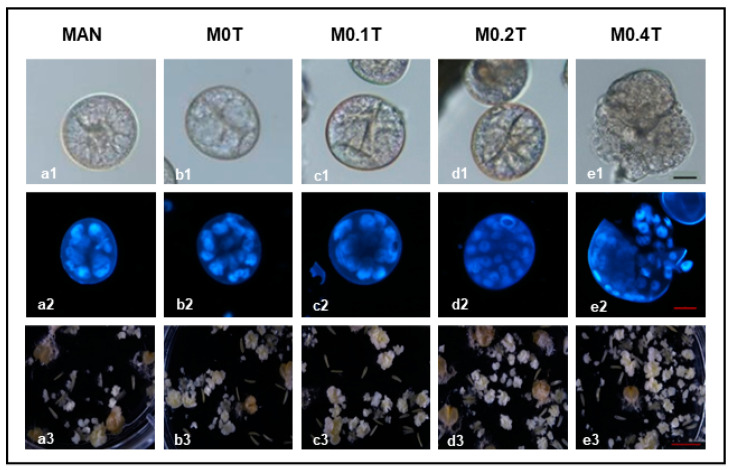
Microspore embryogenesis of bread wheat after the application of different concentrations of trichostatin A (TSA) simultaneously with mannitol stress treatment. Multicellular structures (enclosed by the exine) and proembryos (multicellular structures released from the exine) (**a1**–**e1** and **a2**–**e2**) at 10 days of culture and mature embryos and callus-like structures (**a3**–**e3**) at 40 days of culture from cv. Pavon. **a1**–**e1**: bright field images; **a2**–**e2**: dark field images of multicellular structures and proembryos stained with DAPI. **a1**–**a3**) MAN = 0.7 M mannitol; **b1**–**b3**) M0T = 0.7 M mannitol with 1% DMSO; **c1**–**c3**) M0.1T = mannitol with 0.1 µM TSA; **d1**–**d3**) M02T = mannitol with 0.2 µMTSA; **e1**–**e3**) M0.4T = mannitol with 0.4 µMTSA. Scale bars for a1–e1 and a2–e2 = 20 µM; scale bars for a3–e3 = 2.5 mm.

When TSA was applied simultaneously with MAN, a great effect of TSA on the number of proembryos was observed. The highest number was achieved with 0.4 µM TSA (M0.4T), rendering a 3-fold increase compared with MAN or M0T (mannitol with 1% DMSO) (Table 1 option D). After 10 days of culture, compact multicellular structures enclosed by the exine were observed in all treatments (Figure 1a1–e1), but only in M0.4T, some of the multicellular structures were released from the exine giving rise to a proembryo (Figure 1e1). After DAPI staining, multicellular structures and/or proembryos with more than 20 nuclei were visible in both M0.2T and M0.4T (Figure 1d2–e2), whereas structures with a smaller number of nuclei were observed in controls (MAN and M0T) and M0.1T (Figure 1a2–c2). The M0.2T and M0.4T treatments also produced up to a 2.2 times higher number of embryos and green plants than MAN (Table 1 option D and Figure 1a3,d3–e3).

Nevertheless, it should be highlighted that the control M0T yielded around a 1.5 times higher number of embryos (Figure 1a3,b3) and green plants than MAN (Table 1 option D). Significant differences in the percentage of spontaneous doubling were observed, mainly due to the low value in MAN. DMSO (M0T) produced a 1.7-fold increase in this variable, which was only slightly raised up to 2-fold in M0.4T (Table 1 option D). Altogether, the M0.4T treatment produced a 4- and almost 2 times higher number of green DH plants than MAN and M0T (24.9, 5.9, and 14.7, respectively).

Each wheat cultivar exhibited a different response to the simultaneous treatment of TSA with MAN, in terms of both optimal concentration and magnitude of the effect caused (Table 2). In the high-responding cv. Pavon, similar numbers of proembryos, embryos, and green plants were obtained with M0.2T and M0.4T, which were 2–2.5 times higher than those of MAN. Interestingly, the M0.4T treatment also yielded the largest number of proembryos, embryos, and green plants in the medium-low responding cv. Caramba, with a 5.5-, 3.2-, and 3.6-fold increase, respectively, compared with MAN. It should be noted that the DMSO control treatment (M0T) increased the number of proembryos by 77% in Caramba, but it had no effect on Pavon. Unexpectedly, the number of proembryos obtained with the M0.4T treatment in Caramba was 30% higher than in Pavon. Regardless, the number of embryos in both cultivars was similar, indicating that a high percentage of proembryos did not develop a mature embryo in Caramba. The effect of TSA treatment on the percentage of spontaneous doubling also depended on the cultivar (Table 2). In Caramba, M0.2T significantly enhanced the percentage of doubling from approximately 55% obtained in MAN and M0T to 79.3%. However, M0T caused the major increase in this variable from 28% in MAN to 52.7%, and only M0.4T slightly raised the percentage of doubling up to 58.9% in Pavon.

### 2.3. Application of TSA as a Unique Induction Treatment

**Figure 2:**
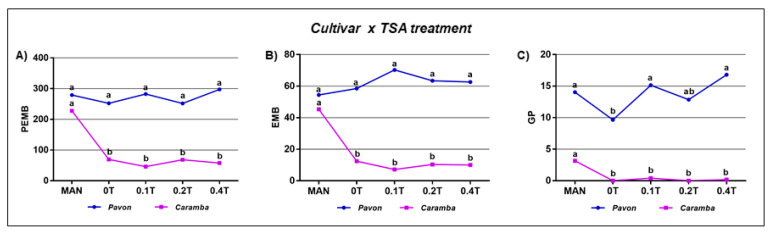
Microspore embryogenesis of bread wheat cvs. Pavon and Caramba after the application of different concentrations of TSA for 2 h as a unique induction treatment before culture. (**A**) Analysis of the interaction cultivar x treatment for the variables: (**A**) Number of proembryos (PEMB); (**B**) Number of embryos (EMB); (**C**) Number of green plants (GP), all per 100 anthers. Treatments with the same letter within each variable and cultivar are not significantly different (*p* ˂ 0.05) according to a Duncan test. MAN = 0.7 M mannitol for 5 days at 25 °C (control cultures); 0T = 1% DMSO; 0.1T = 0.1 µM TSA; 02T = 0.2 µM TSA; 0.4T = 0.4 µM TSA.

TSA treatments at 0, 0.1, 0.2, and 0.4 µM (0T, 0.1T, 0.2T, and 0.4T) were assayed for 2 h in fresh anthers as a unique induction treatment before culture and compared with MAN (Supplementary Figure S1E and Table 1 option E). A significant interaction cultivar × TSA treatment was observed for the numbers of proembryos, embryos, and green plants, indicating that the two cultivars responded differently to TSA treatments. In Pavon, a similar number of proembryos, embryos, and green plants were obtained for all TSA treatments and controls (Figure 2A–C), except for the number of green plants in the control 0T (DMSO), in which a significant reduction with respect to MAN (9.6 and 14.4, respectively) was observed. In Caramba, all treatments produced an almost three times smaller number of proembryos, embryos, and green plants per 100 anthers than MAN (Figure 2A–C).

Interestingly, the percentages of green plants and spontaneous chromosome doubling were affected similarly by TSA in both cultivars (Table 1 option E). The highest percentage of green plants was achieved in MAN (64%), which was reduced 2-fold in 0T. Only 0.1T produced similar values for this variable to MAN. However, the percentage of spontaneous chromosome doubling in 0T was twice that in MAN (65.6% and 31.1%, respectively) and increased up to 85.3% with 0.2T treatment. Finally, the number of green DH plants obtained with 0.1 to 0.4 µM TSA was doubled than with MAN.

### 2.4. Microspore Morphological Changes Associated with ME Induction and TSA 

To determine the morphological changes associated with ME induction with or without TSA, isolated microspores before (FM) and after mannitol (MAN) and mannitol with 0.4 µM TSA (M0.4T) (the most efficient treatment for ME induction in our study) were microscopically characterized.

**Figure 3:**
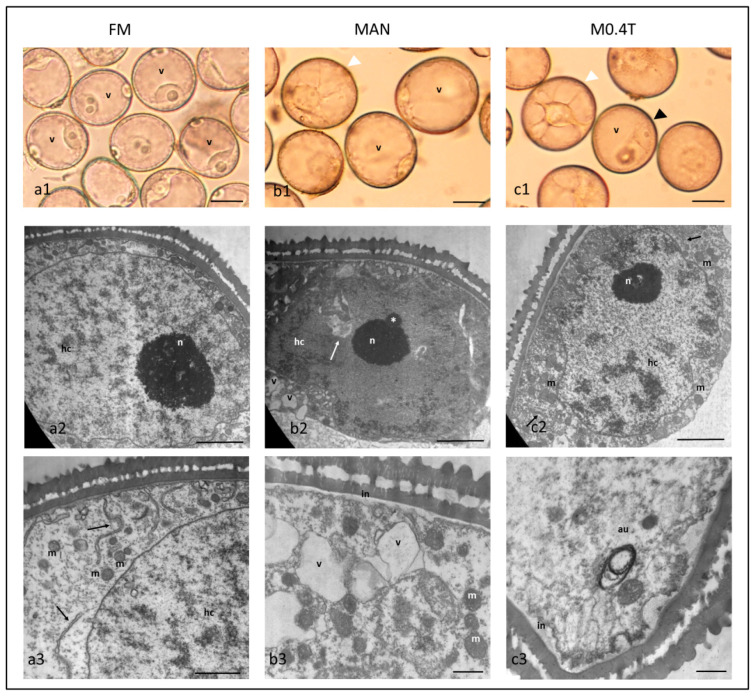
Morphological and ultrastructural characterization of freshly isolated microspores of bread wheat cv. Pavon (FM) (**a1**–**a3**), after 0.7 M mannitol (MAN) (**b1**–**b3**) and 0.7 M mannitol with 0.4 µM TSA treatments (M0.4T) (**c1**–**c3**). FM showed a large vacuole (v), a euchromatin nuclei with small patches of heterochromatin (hc), and a prominent nucleolus (**a1**–**a2**); cytoplasm with abundant free ribosomes, mitochondria (m), and RER (black arrows) (**a3**). Both MAN and M0.4T showed larger microspores, some with SLM morphology (**b1, c1,** white arrowheads) or symmetrically divided (**c1**, black arrowhead); a euchromatic nucleus with patches of heterochromatin (hc) (**b2, c2**), a small nucleolus with a Cajal body (asterisk) (**b2**), and invaginations of the nuclear envelope (**b2**, white arrow); cytoplasm with vacuoles (v), free ribosomes, mitochondria (m), and autophagosome (au) (**b2, b3, c3**); and an intine layer (in) (**b3, c3**). Scale bars for **a1**–**c1**= 20 µm; scale bars for **a2**, **b2**, **c2** = 2 µm; scale bars for **a3** = 1 µm; and scale bars for **b3**, **c3** = 0.5 µm.

Microspores isolated from fresh anthers were mainly in the late uninucleate stage, with a nucleus containing one prominent nucleolus or two smaller nucleoli, frequently located at the opposite side of the pore (Figure 3a1). After treatment with MAN or M0.4T microspores increased in size, and in some microspores, the vacuole was fragmented by cytoplasmic strands giving rise to the typical ‘star-like’ morphology (SLM), whereas others underwent a symmetrical division (Figure 3b1,c1). A larger proportion of these two types of microspores were observed in M0.4T than in MAN (24% and 15%, average of five microspore isolations, data not shown).

The ultrastructure of late uninucleate microspores showed nuclei with a decondensed chromatin pattern with small and homogeneously distributed patches of heterochromatin (Figure 3a2). The cytoplasm contained abundant free ribosomes, mitochondria and rough endoplasmic reticulum (RER) (Figure 3a3).

After the treatments, the microspores ultrastructure changed and diverse types of microspores according to the nuclear and cytoplasm morphology could be observed, but a specific type of microspore could not be associated with MAN or M0.4T. Some nuclei showed completely decondensed chromatin, while others presented heterochromatic patches (Figure 3b2,c2) resembling chromosomes attached to the nuclear envelope (Figure 3c2). Nucleoli of smaller size than in fresh microspores were observed, occasionally with Cajal bodies (Figure 3b2). In both treatments, nuclear envelope invaginations containing cytoplasm were shown (Figure 3b2). Some microspores presented a cytoplasm with abundant mitochondria, undifferentiated plastids, free ribosomes and RER (Figure 3b2,c2). Others had a small number of organelles, some vacuoles and multilamellar structures, probably autophagosomes (Figure 3c3). Vacuoles and membranous elements with varied degrees of luminal density were observed after both treatments (Figure 3b2,b3,c3), some of them frequently associated with irregularities of the cell wall and the presence of low electro-dense fibrillar material deposited in the intine layer (Figure 3b3,c3).

### 2.5. Transcript Level Changes in Marker Genes for Early and Middle Stages of Microspore Embryogenesis

The expression of marker genes for wheat ME [25] was compared between mannitol with 0.4 µM TSA (M0.4T) and 0.7 M mannitol (MAN). The analysis was performed by quantitative RT-PCR using isolated microspores from cvs. Pavon and Caramba after 0, 3, and 5 days of anther treatment (0dT, 3dT, and 5dT) and 3 and 7 days of culture (3dC and 7dC).

**Figure 4:**
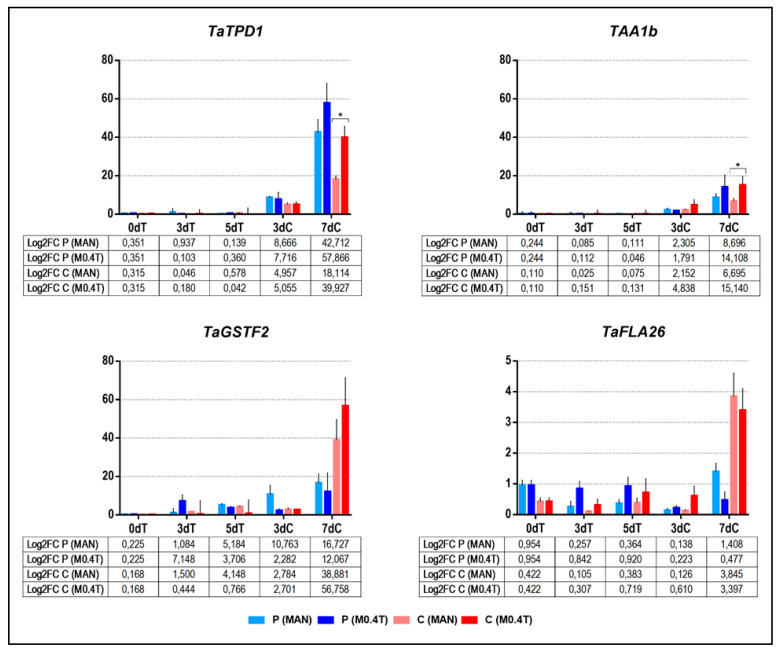
qRT-PCR analysis of early (*TaTPD1*, *TAA1b* and *TaGSTF2*) and middle (*TaFLA26*) marker genes for bread wheat microspore embryogenesis after application of treatments with 0.7 M mannitol (MAN) and mannitol with 0.4 µM TSA (M0.4T) in cvs. Pavon (P) and Caramba (C) after 0, 3, and 5 days of treatment (0dT, 3dT, and 5dT) and 3 and 7 days of culture (3dC and 7dC). The y-axis shows the log2 fold-change when the data was normalized using *Ta27771* as endogenous gene control. Error bars represent the standard error of the means. Asterisk indicates significant differences between treatments for each cultivar, according to a Student´s *t*-test (*p* ˂ 0.05).

The marker genes for early stages of embryonic development, *TaTPD1* (*TAPETUM DETERMINANT 1*, TraesCS4D02G104300) and *TAA1b* (fatty acyl-CoA reductase, *TRITICUM AESTIVUM ANTHER-SPECIFIC 1b*, TraesCS4D02G008300), showed an increase in the transcript level at 3dC that was significantly higher at 7dC in both treatments and cultivars (Figure 4). Differences in transcript levels were observed between MAN and M0.4T in early stages of culture. The M0.4T increased the level of both genes in the two cultivars at 7dC, although only significantly in Caramba. In both treatments, the level of *TaTPD1* in Caramba was lower than in Pavon, whereas similar levels of *TAA1b* were observed in the two cultivars. The transcript level of the early gene *TaGSTF2* (*GLUTATHIONE TRANSFERASE F2*, TraesCS1D02G094900) was low during stress treatments and early culture (3dC), but was highly induced at 7dC. At this stage, the transcript level was higher in Caramba than in Pavon in both treatments, but cultivars responded differently to TSA, increasing in Caramba and decreasing in Pavon. The middle marker gene *TaFLA26* (*FASCICLIN-LIKE ARABINOGALACTAN PROTEIN 26*, TraesCS5B02G428500) was expressed at lower level than the other genes. The highest transcript level was shown after 7dC in MAN. *TaFLA26* was significantly downregulated with M0.4T compared with MAN in Pavon but not in Caramba.

## 3. Discussion

Systems based on the modification of the developmental program of cells, organs, or plants offer an enormous potential in plant biotechnology and provide valuable materials for plant breeding. In ME, the application of a stress treatment to mid-to-late uninucleate microspores triggers a complex response, resulting in the development of an embryogenesis pattern that allows the production of DH plants (for a review, see [26]). However, one of the main drawbacks in ME is the limited number of microspores capable of reprogramming their developmental pathway in response to external stimuli and producing embryos [27].

Among the different stress treatments tested to induce ME in wheat, cold or starvation were the most frequently applied [8]. Therefore, a cold treatment of 7–9 day at 4 °C (CLD) and starvation plus osmotic treatment with 0.7 M mannitol for 5 days at 25 °C (MAN) were used in this study. Microspores responded differently to the two stresses, affecting mainly the percentages of green plants and chromosome spontaneous doubling (Table 1). In our hands, mannitol treatments rendered between 3.6 and 5.4 higher number of green plants than cold. Similar results were reported in barley when contrasting 28 days of cold and 4 days of 1.0 M mannitol [28]. Nevertheless, the rates of spontaneous doubling achieved with cold treatments were almost 3 times higher than with mannitol and more than twice than those obtained with a similar cold treatment in wheat [6]. As a consequence, the number of green DH plants obtained in our study with MAN was 2–4.5 times larger than with CLD (Table 1 options A–D).

### 3.1. TSA Application Simultaneously with Mannitol Treatment Increases Microspore Embryogenesis

Promising strategies for ME induction are based on the application of trichostatin A (TSA), a canonical histone deacetylases inhibitor, whose inhibitory effect over HDACs has been widely demonstrated in the experimentation of human diseases [29,30] but also in plants, such as in maize mitosis or *Brassica* ME [20,31]. However, other nonhistone proteins were identified in various compartments as substrates of plant HDACs (for a review, see [32]). The effect of TSA in ME depended on the concentration, duration, and time of application (stress treatment or culture) and their combination with stress treatment in *Brassica*, barley, and wheat [20,21,22,23,24]. In this study, TSA was applied as a unique induction treatment as well as in different combinations with cold and mannitol stress treatments. The effect of the TSA solvent (DMSO) was also evaluated.

In our initial approach, we considered that the first report describing the effectiveness of TSA in ME of bread wheat was performed with a short application of TSA (10 min) to isolated microspores after cold stress, attaining a significant increase in the number of green plants in 3 out of 8 genotypes and F1 [23]. In our assay, the application of TSA to anthers after a cold or mannitol treatment was done for 2 h to allow the absorption by the microspores. However, our results with 0.1–0.4 µM TSA after cold treatment did not further enhance the efficiency of ME (Table 1 option A). These contradictory results could be due to the different conditions used in both studies. The application of TSA after a mannitol treatment has only been reported in barley [22] where 0.5 µM TSA for 24 h to isolated microspores, after a starvation treatment, led to a complete inhibition of ME. Surprisingly, in our study, 0.1 µM TSA applied to anthers for 2 h after mannitol slightly increased the percentage of spontaneous doubling, resulting in a 1.6-fold increase in the number of green DH plants compared with MAN in both cultivars (Table 1 option B).

Since ME efficiency was slightly increased with the first strategy, we decided to evaluate the application of TSA simultaneously with the stress treatments. Previously, successful application of 0.5 µM TSA for 20 h had been reported with a 33 °C treatment in *Brassica* [20]. However, a TSA treatment as long as 5 days in MAN or 7–9 days with CLD had not been previously tested in any species. Our results showed that 0.4 µM TSA simultaneously with CLD or MAN (C0.4T and M0.4T, respectively) was the best strategy to increase ME efficiency in bread wheat (Table 1 options C, D). Thus, TSA with CLD resulted in a 3.2- and a 2.5 times higher number of green plants and green DH plants than CLD. A greater effect was observed when TSA was applied with MAN, resulting in a 4-fold increase in the number of green DH plants, mainly because TSA doubled the number of green plants and the percentage of spontaneous doubling compared with MAN. DMSO did not have an effect on this variable with CLD, whereas its value was doubled with MAN. Our results agree with the increased number of proembryos obtained in barley when 0.5 µM TSA was applied during the first 24 h of mannitol treatment in the high-responding cv. Igri [22].

One of the main drawbacks of wheat ME is the genotype dependency [6,33]. Even previous results with TSA have shown that each genotype has an optimal dose of TSA in wheat and *Brassica rapa* [21,23]. Surprisingly, the same treatment (M0.4T) was the most efficient for the two cultivars in our study (Table 2). However, TSA caused a larger effect on the number of proembryos in the medium-low-responding cv. Caramba than in cv. Pavon (5.5-fold and a 2.4-fold increase, respectively, with respect to MAN). Nevertheless, as described in *Brassica* [20], most proembryos resulted in callus-like structures that did not regenerate plants. Despite this, the number of green plants obtained was 6 times and 4 times higher than MAN in Caramba and Pavon, respectively.

Due to the observed effect of TSA when applied simultaneously with stress treatment, we wondered whether TSA alone was able to promote ME. When this strategy was assayed in *Brassica*, a higher concentration was required, and the rates of ME were lower than those with TSA with a heat treatment [20]. Our results indicate that the application 0.4 µM TSA to fresh anthers for 2 h could be used as an alternative to MAN treatment only in the high-responding cv. Pavon (Figure 2), with up to a 3-fold increase in the number of green DH plants (data not shown). According to observations in studies with *Brassica* [20,21], a higher concentration or longer application of TSA could be necessary to induce ME in low-responding cultivars. The requirements of more severe stress treatments in low-responding cultivars have also been described previously in barley and wheat [24,28]. Therefore, new strategies that combine TSA with stress treatments could be tested as an alternative approach for ME induction in bread wheat, but the number of green DH plants per 100 anthers need to be evaluated to demonstrate their effectiveness. In wheat, two strategies for TSA application to isolated microspores from 21 to 28 days cold-stressed spikes were compared: 0.1 µM TSA for 10 min during the isolation procedure and 0.01 µM TSA in the culture medium [24]. Although the second strategy rendered a 2- to 3.5-fold increase in the number of green plants in two cultivars of wheat, 70–88% of plants were haploids. In our hands, a shorter cold treatment (7–9 days) in combination with any of the two strategies for TSA application produced a high percentage of spontaneous doubling (60–80%) and a high percentage of albino plants (80–90%) (Table 1). This rate of spontaneous doubling was also observed when 0.4 µM TSA with mannitol (62%) was applied. These rates are high enough to avoid colchicine treatment for obtaining DH populations from F1s, thus saving time and cost in plant breeding programs. Therefore, the strategy of applying 0.4 µM TSA during a cold (7–9 days at 4 °C) and a 0.7 M mannitol (5 days at 25 °C) treatment could be very helpful to those laboratories using anther culture methodology for wheat DH production.

The TSA solvent (DMSO) causes a contradictory effect on the induction of ME, depending on the strategy used and the genotype. Although DMSO has been widely used as solvent, a few studies have been performed to examine its impact on plant systems, and the results are also contradictory. A toxic effect has been described in rice seedlings [34], whereas there was no reaction in shoot growth when used as colchicine solvent in chromosome doubling treatments [35]. In ME, a beneficial effect of 1% DMSO with a 0.7 M mannitol treatment, which was greater in low-responding cultivars, has been reported in barley and wheat [36]. However, in barley, 24 h of 0.015% DMSO with a mannitol treatment drastically reduced the viability of microspores and proembryo formation [22]. Nevertheless, the effect of DMSO and TSA has not been studied separately in previous reports in wheat ME [23,24]. In our study, the major positive effect of DMSO was observed when it was applied simultaneously with mannitol, with a 2.5 times higher number of green DH than MAN (Table 1 option D). DMSO had the greatest impact not only on the number of proembryos in Caramba but also on the percentage of spontaneous doubling in Pavon. This variable was also enhanced significantly with DMSO when applied for a short time as a unique treatment in Pavon. These differences could reflect the effect of DMSO on different processes due to its ability to induce cell reprogramming through changes in the epigenetic landscape [37,38] or by enhancing the permeability of Ca^2+^ [39] that is known to increase ME in barley [40].

Altogether, we obtained a 4-fold increase in the number of green DH plants compared with MAN when 0.4 µM TSA was applied with 0.7 M mannitol for 5 days. This enhancement of ME was truly a consequence of the combined effect of TSA and DMSO. Interestingly, TSA also influences the Ca^2+^ gradient, as does DMSO, and disrupts actin filaments in pollen [41]. Elevation of intracellular Ca^2+^ levels and changes in cytoskeleton has also been described in embryogenic microspores of *Brassica* [42,43].

### 3.2. TSA with Mannitol and Mannitol Treatments Trigger Similar Reprogramming Morphological Changes

Two morphological events have drawn attention to the embryogenic response with the most efficient treatment with TSA (M0.4T): the increase in the number of proembryos and the low conversion to mature embryos in both cultivars. Therefore, we wondered whether the reprogramming of microspores after treatment with TSA presented particular morphological changes that we could associate with those characters. To our knowledge, only one study of morphological characterization after a TSA treatment during heat stress has been carried out in the multicellular structures at 5 days of culture in *Brassica* by DAPI staining [20].

In this study, after MAN or M0.4T treatments, we identified a higher number of microspores that acquired SLM morphology or divided symmetrically in M0.4T. This difference could determine the greater number of embryos and callus-like structures observed in this treatment (Figure 1a3–e3). SLM morphology or a symmetric microspore division has been associated with embryogenic potential in wheat, rice, and tobacco, among others [44,45,46], although these characters are not always a reliable marker for embryogenesis [47,48].

Ultrastructural characterization of the microspore contributed to associate the morphological characters of its nucleus and cytoplasm to its functional state. Fresh late uninucleate microspores showed characteristics of high transcriptional activity, such as a decondensed chromatin with patches of heterochromatin distributed homogeneously and a prominent nucleolus (Figure 3a2), as described previously [49,50]. Additionally, a high degree of cytoplasmic activity was observed, as revealed by the large number of free ribosomes, RER, and mitochondria (Figure 3a3), in accordance with Sharma et al. [50].

Our results indicate that the application of TSA simultaneously with mannitol (M0.4T) or mannitol treatment (MAN) induced similar ultrastructural changes in the microspore reprogramming. Similar results were reported with a TSA treatment and heat stress in *Brassica*, in light microscopy studies [20]. Therefore, it could be that pathways controlled by acetylation of histones and/or other proteins also underlie the microspore response to mannitol stress, as suggested by Li et al., 2014 [20] with a heat treatment.

After treatments, the microspores showed characteristics of high transcriptional activity, as in a general response to stress, involving adjustment of development to increase stress tolerance. Thus, different degrees of chromatin condensation, an increased number of Cajal bodies, small nucleoli, and invaginations of the nuclear envelope were observed (Figure 3b2,c2). This morphology has been reported not only in ME induction in tobacco and *Brassica*, among others, but also in stressed cells of barley and wheat [49,51,52,53,54]. A heterogeneous population, in terms of cytoplasm, including both microspores with an organelle-rich cytoplasm without starch deposits and microspores with a small number of organelles and the presence of vacuoles, membranous elements, and autophagosomes were also observed after the two treatments (Figure 3b3,c3). The presence of a heterogenic population after stress treatment has been reported in barley [48]. Microspores with degenerated cytoplasm and replacement with new organelles have been described in ME of tobacco, maize, *Brassica*, barley, and bread wheat ME [47,51,55,56]. In *Brassica*, this cytoplasmic cleaning seems to be mediated by autophagy-related elements [55], and the presence of autophagy processes have also been described in barley [57]. Therefore, after both treatments, the microspores identified with a low number of organelles, vacuoles, membranous elements, and autophagosomes could follow the embryogenic pathway, whereas microspores with a rich cytoplasm could represent a gametophytic development. As far as we know, this is the first study showing autophagy could be involved in ME induction in bread wheat.

### 3.3. TSA Modifies the Transcript Level of Marker Genes for Early Microspore Embryogenesis

Little information is available about the effect of TSA on the expression of genes associated with ME. An only report has indicated that TSA treatment is sufficient to activate embryo gene expression in *Brassica* [20].

*TaTPD1* and *TAA1* had been described as early markers of wheat ME, as they were induced at 5dC in anther culture after a 0.7M mannitol treatment [25]. This study confirmed its status as early markers, since they were induced at 3dC in two cultivars of different androgenic response (Figure 4). Our results also show that TSA (M0.4T) does not modify the induction pattern over time from that observed after MAN. However, TSA increased the transcript level of both genes. Although these genes are probably implicated in intraembryogenic structure communication [25], they have been associated with different mechanisms. *TPD1* was first described as being involved in signaling of tapetum cell fate in *Arabidopsis* [58]. In addition, a *TPD1-like* gene is a marker for early zygotic reprogramming in wheat [59], and is involved in cell proliferation by regulating auxin signaling and cell cycle genes [60]. *TAA1b* encodes a fatty acyl-CoA reductase (FAR) [61] that expresses specifically in anthers during microspore wall thickening [61,62] and that could be regulated by MYB transcription factors [63]. In this sense, auxin, G1-to-S phase transition-related genes, and MYB genes were also upregulated immediately after TSA treatment in *Brassica* ME [20] and somatic embryogenesis [64]. Therefore, the early ME signaling pathways in which *TaTPD1* and *TAA1* participate could be mediated by the effect of TSA on the acetylation of HDACs or other proteins.

*TaGSTF2* is a member of a major group of detoxification enzymes in wheat [65,66]. Its expression has been associated with cell protection, enhanced viability, and reprogramming efficiency in ME of triticale and wheat [18,20]. Although it is an early marker for ME in wheat [20], our results do not register an apparent increase at 3dC, unlike *TaTPD1* and *TAA1*. The transcript level of this gene at 7dC, after MAN or M0.4T, seems to indicate that the medium-responding cv. Caramba requires greater protection than Pavon during early stages of culture (Figure 4). It also seems that a higher level of protection is needed during reprogramming and proliferation activity, ending with the proembryo formation induced by TSA treatment. Finally, the *TaFLA26* gene was studied as a ME middle-stages marker, since it is induced between 5 and 10 days of culture [20]. *TaFLA26* encodes a fasciclin-like arabinogalactan protein (FLAs), a subclass of AGPs [67]. The role of AGPs in ME has been widely reported, and even AGPs have been identified as early markers of ME in *Brassicaceae* [68,69,70]. However, the lower levels of the transcript with TSA treatment in both cultivars indicate that this gene does not seem to perform a prominent role during multicellular structure formation.

Chromatin modifications play a fundamental role in developmental changes in response to stress. In this study, we have applied a histone deacetylase inhibitor (TSA) to increase the efficiency of microspore reprogramming towards an embryogenic pathway. Microspores responded differently to diverse strategies of TSA application: as a unique treatment, after stress treatment, or simultaneously with cold or mannitol treatment, in two cultivars of bread wheat with a different ME response. The highest efficiency was achieved with 0.4 µM TSA and mannitol treatment simultaneously, which rendered 4 times higher number of green DH plants than mannitol. The application of 0.4 µM TSA during 7–9 days cold treatment could also be valid for increasing ME, but at a lower rate. However, TSA as a unique treatment was only effective in the high-responding cultivar. The morphological and ultrastructural characterization revealed that morphological changes occurring at the early steps in microspore reprogramming after induction with TSA and mannitol were similar to those observed with mannitol. However, TSA with mannitol increased the number of SLM and embryogenic microspores. The changes in transcript level of the ME markers produced by TSA treatment suggest that, directly or indirectly, modifications in the patterns of acetylation are associated with initial signaling pathways. However, further studies should be undertaken to clarify the molecular mechanisms underlying the induction of wheat microspore embryogenesis by TSA.

## 4. Materials and Methods

### 4.1. Material, Growing Conditions of Donor Plants, and Harvesting of Spikes

The spring bread wheat cultivars Pavon and Caramba were used for anther culture. Pavon has a high ME response, whereas Caramba has a medium-low response. Donor plants were grown as described by Soriano et al., 2007 [71]. For anther culture, spikes inside the sheath were sprayed with 96% ethanol for sterilization. Anthers containing the majority of microspores at the mid- to late-uninucleate stage, determined by DAPI (4′,6-Diamidine-2’-phenylindole dihydrochlorid) staining, were excised. Ovaries from Caramba flowers that contained microspores in the late binucleate stage of development were used for ovary preconditioned medium. Spikes out of the sheath were directly sprayed with ethanol before culture.

### 4.2. Preparation of Ovary Preconditioned Medium and Ovary Coculture (OVPCM)

Thirty ovaries excised from flowers containing microspores at the late binucleate stage were cultured in 10 mL of MS3M medium [72], a MMS3 medium [73] containing 1 mgL^−1^ 2, 4-dichlorophenoxyacetic acid (2,4-D) and 1 mg/L benzyladenine (BA) supplemented with 200 gL^−1^ Ficoll type 400, in a 6-cm Ø Petri dish. Cultures were maintained in the dark at 25 °C for 5 days before use and ovaries were maintained in medium during the first 40 days of culture (OVPCM) [72]. To avoid variability between treatments, OVPCM from the same Petri dish was used for the five treatments from the same replicate, and thus, 2 mL of medium and 6 ovaries were randomly distributed in the 5 Petri dishes of 3 cm Ø corresponding to one replicate.

### 4.3. Application of the Histone Deacetylase Inhibitor Trichostatin A (TSA)

The effect of TSA (Sigma T8852) on wheat ME was assayed in combination or not with different stress treatments. Stock solutions of 5, 10, and 20 µM TSA in 50% DMSO were prepared. For 0.1, 0.2, and 0.4 µM TSA treatments, 20 µL from each stock solution were added per milliliter of medium (0.1T, 0.2T, and 0.4T, respectively, final concentration of DMSO in the medium 1%). To evaluate the effect of DMSO, a treatment containing 1% DMSO (0T) was additionally included in all experiments.

#### 4.3.1. Application of TSA after Cold Stress Treatment

Freshly harvested spikes covered with aluminum foil were maintained in a flask container with water at 4 °C for 7–9 days. After the cold treatment, anthers were excised and randomly distributed in 3-cm Ø Petri dishes containing 2 mL of MS3M liquid medium (control cold, CLD) or MS3M liquid medium with 0 (DMSO), 0.1, 0.2, and 0.4 µM TSA (C + 0T, C + 0.1T, C + 0.2T, C + 0.4T). After 2 h of DMSO or/and TSA treatment at room temperature (RT) in the dark, the medium was removed from all treatments (including controls). Anthers, in each Petri dish, were washed for 5 min with MS3M liquid medium to remove DMSO or/and TSA, and afterwards were cultured in OVPCM. Fifteen replicates per cultivar were assessed (Supplementary Figure S1A).

#### 4.3.2. Application of TSA after Mannitol Stress Treatment

For mannitol treatment, freshly excised anthers were randomly distributed in 5 Petri dishes (one replicate) of 6-cm Ø containing 0.7 M mannitol, 5.9 g L^−1^ CaCl_2_ 2H_2_O plus macronutrients from FHG medium [74] solidified with 8 g L^−1^ Sea Plaque agarose [75] and kept for 5 days at 25 °C in the dark. After the mannitol treatment, anthers from each dish from the same replicate were transferred to 3-cm Ø Petri dishes containing MS3M liquid medium (control mannitol, MAN) or MS3M liquid medium with 0 (DMSO), 0.1, 0.2, and 0.4 µM TSA (M + 0T, M + 0.1T, M + 0.2T, and M + 0.4T). After 2 h at RT in the dark, the medium was removed from all treatments (including controls) and anthers in each Petri dish were washed with MS3M liquid medium for 5 min and cultured in OVPCM medium. Twenty-two replicates from Pavon and 18 from Caramba were assessed (Supplementary Figure S1B).

#### 4.3.3. Application of TSA in Combination with Cold Stress Treatment

Freshly harvested spikes, inside the sheath, were randomly distributed in Falcon tubes containing 7 mL of water with 0 (DMSO), 0.1, 0.2, and 0.4 µM TSA (C0T, C0.1T, C0.2T, and C0.4T, respectively) or water (cold control, CLD). The Falcon tubes were covered with aluminum foil and placed at 4 °C for 7–9 days. After treatment, the basal part of the spikes from all treatments was washed with water for 5 min before sterilization. Anthers from each spike were excised and cultured in a 3-cm Ø Petri dish with 2 mL of OVPCM medium (Supplementary Figure S1C). Twenty replicates from each cultivar were cultured.

#### 4.3.4. Application of TSA in Combination with Mannitol Stress Treatment

Freshly excised anthers were randomly distributed in Petri dishes with solidified 0.7 M mannitol stress medium (mannitol control, MAN) or in mannitol with 0 (DMSO), 0.1, 0.2, and 0.4 µM TSA (M0T, M0.1T, M0.2T, and M0.4T, respectively) and placed in the dark at 25 °C. After 5 days of treatment, anthers from each replicate and all treatments (including controls) were plated in 3-cm Ø Petri dishes, washed with MS3M medium for 5 min, and cultured in OVPCM medium. Twenty-eight replicates from Pavon and 17 replicates from Caramba were assessed (Supplementary Figure S1D).

#### 4.3.5. Application of TSA as a Unique Anther Treatment

Freshly excised anthers were randomly distributed on MS3M liquid medium containing 0, 0.1, 0.2, and 0.4 µM TSA for 2 h at RT in the dark (0T, 0.1T, 0.2T, and 0.4T, respectively) or in 0.7 M mannitol stress medium for 5 days at 25 °C in the dark (control culture, MAN). After that, anthers for all treatments were washed with MS3M medium for 5 min and transferred to OVPCM (for control cultures, OVPCM were kept at 4° C for 5 days before culture). Twenty replicates from Pavon and 15 replicates from Caramba were assessed in this experiment (Supplementary Figure S1E).

### 4.4. Anther Culture

Anthers cultured in OVPCM were kept at 25 °C in the dark. After 10–12 days, plates were replenished with 2 mL of MS3M with 400 g/L instead of 200 g/L of Ficoll Type 400. After 40 days, embryos were transferred to J25–8 medium [76] for regeneration. Embryos were kept in the dark at 25 °C for 2 days and then transferred to the light. Ploidy analysis of regenerated plants was performed with a PAS cytometer (Partec-Sysmex, Germany) as described by Soriano et al., 2007 [71]).

### 4.5. Stereoscopic and Microscopic Observation, DAPI Staining, and Ultrastructural Studies

Anther cultures were visualized under an inverted Nikon Eclipse-T300 and a SMZ750 Nikon binocular stereoscope. Images from isolated microspores, proembryos and embryos were recorded by digital cameras (Digital sight DS-U2 and Digital sight DS-Fi1) and processed using NIS-Elements D (AR 2.10 Laboratory Imaging System, Ltd.).

Microspores and proembryos were isolated following the protocol described by Castillo et al., 2000 [77]. For DAPI analysis, proembryos were isolated from anthers treated with TSA in combination with mannitol stress treatment for 5 days and cultured for 10 days. Nuclei were stained with 0.4 µg/mL DAPI (4′,6-Diamidine-2’-phenylindole dihydrochlorid) for 10 min before observation under epifluorescence and filters EX 365/DM 400/BA.

For ultrastructural analysis, microspores from Pavon were isolated from fresh anthers and anthers treated with 0.7 M mannitol (MAN) and 0.7 M mannitol plus 4 µM TSA (M0.4T) for 5 days. Microspores were fixed in Karnovsky’s fixative (4% paraformaldehyde and 5% glutaraldehyde) in 0.1 M cacodylate buffer pH 7.3 for 5 h at RT. After two washes with the same buffer, microspores were postfixed for 2 h at RT with 1% OsO_4_ in PBS. After three washes in PBS, microspores were pre-embedded in 2% agarose. Samples were dehydrated in an ethanol series at 4 °C and embedded in Unicryl following the manufacturer´s instructions. Polymerization was performed at −20 °C with UV light for 54 h. Semithin sections (1 µm thick) were cut for light microscopy. For the ultrastructural investigation, suitable ultrathin sections were selected, double stained with uranyl acetate and lead hydroxide, and examined with a Libra 120 (Carl Zeiss NTS GMBH, Oberkochen, Germany).

### 4.6. Transcript Level Analysis by Quantitative RT-PCR

Marker genes for early and middle stages of embryo development in wheat ME were selected from previous studies (early and middle genes induced at 5 or 10 days of culture, respectively) [25]: *TaTPD1*, *TAA1b*, *TaGSTF2*, and *TaFLA26*. The original sequence of the marker genes based on HarvEST: Wheat v. 1.59 was subjected to BLASTN against *Triticum aestivum* IWGSC (Genomic sequence) in EnsemblPlants (http://plants.ensembl.org/Triticum_aestivum) for identification of overlapping genes.

For transcript level analysis, microspores of Pavon and Caramba of three independent biological replicates were isolated following the protocol described by Castillo et al. 2000 [77] after 0, 3, and 5 days of anther treatment in 0.7 M mannitol (MAN) or in 0.7 M mannitol containing 0.4 µM TSA (M0.4T) (0dT, 3dT, and 5dT, respectively) and after 3 and 7 days of culture in OVPCM medium (3dC and 7dC, respectively). Isolated microspores were frozen in liquid nitrogen, and total RNA was isolated using TRIzol Reagent (Gibco BRL) and passed through RNeasy columns (Qiagen) for further clean up. Double-stranded cDNA was synthesized using the M-MLV Reverse Transcriptase kit from Promega. Real-time qPCR reactions were performed with Fast SYBR Green Master Mix (Applied Biosystems) and Rox (ROX Reference Dye, Invitrogen). The reaction conditions were optimized at: 95 °C for 10 min, followed by 40 cycles of 95 °C for 15 s and 60 °C for 1 min, in the PCR7500 Fast Real-Time PCR System (Applied Biosystems), using the *Ta.27771* gene as a reference [78]. Data from the qPCR reactions were analyzed using the Livak (2^-ΔΔCT^) calculation method [79]. For the calculation of the standard errors, the values of the relative expression level (2^-ΔΔCT^) were used. For statistical analysis, the values of 2^-ΔΔCT^ were transformed with log2 to correct the heterogeneity of variance, and the significance of the transformed values was calculated with the Student´s *t*-test.

### 4.7. Statistical Analysis

All experiments were performed with at least two different batches of plants grown in the same growth chamber with the identical growing conditions [71] and different dates of sowing. Each experiment consisted of 15–28 replicates/treatment of 36 anthers each, established in a completely randomized design. When TSA was applied with cold treatment, spikes were randomly distributed among treatments, and anthers from the 6 central flowers of each side of one spike were cultured in the same Petri dish. In the remaining experiments, anthers from the 6 central flowers from each side of 5 spikes were randomly distributed among the five treatments (control cultures, 0, 0.1, 0.2, and 0.4 µM TSA) within replicates.

The following variables were recorded: number of mature embryos (EMB, number of embryos with a well-developed embryo axis per 100 anthers), number of green plants (GP, number of green plants per 100 anthers), percentage of green plants (PGP, number of green plants per 100 total plants), percentage of spontaneous chromosome doubling (PDH, number of doubled haploids per 100 analyzed green plants), and number of green DH plants (GPDH, number of green plants × percentage of spontaneous doubling). The number of proembryos (PEMB, number of multicellular structures that broke the exine per 100 anthers) was calculated as the sum of number of mature embryos and number of calli (a multicellular structure that broke the exine but did not developed an embryo axis). The number of calli was estimated by counting one-tenth of the Petri dish area under a stereoscopic microscope, on a millimeter paper on the 60th day of culture, after all the embryos were transferred for regeneration. Statistical analysis was performed using SAS software (SAS Institute Inc., Cary, NC, and Version 9.1). Normality and homogeneity of variance were tested using Kolmogorov–Smirnoff and Levene’s tests, respectively. Data were transformed applying the square root (x + 0.5) to meet parametric assumptions, except for the percentage of green plants that did not require transformation. The Generalized Linear Model (GLM) procedure was used to conduct ANOVA for all variables except the percentage of spontaneous chromosome doubling and the number of green DH plants, which was analyzed following the FREQ procedure for the Chi square test. The Duncan method (α ≤ 0.05) was used for mean separation.

## Figures and Tables

**Table 1:** Microspore embryogenesis response to trichostatin A (TSA) application, in combination with or without different stress treatment, in two cultivars of bread wheat (Pavon and Caramba). Concentrations of 0, 0.1, 0.2, and 0.4 µM TSA (0T, 0.1T, 0.2T, and 0.4T) were applied in: (A) CLD + 2hTSA: 2 h after a cold treatment, (B) MAN + 2hTSA: 2 h after a mannitol treatment, (C) CLDTSA: 7–9 days during a cold treatment, (D) MANTSA: 5 days during a 0.7 M mannitol treatment, and (E) TSA: 2 h in MS3M liquid medium. TSA was dissolved in 1% DMSO (0T). Control cultures: CLD: spikes were treated at 4 °C for 7–9 days; MAN: anthers were treated on 0.7 M mannitol at 25 °C for 5 days (See Supplementary Figure S1 for detailed information). Table shows TSA treatments means after statistical analysis of the variables: number of proembryos (PEMB), embryos (EMB), green plants (GP), and green DH plant (GPDH), all per 100 anthers, and percentages of green plants (PGP, number of green plants/total plants) and spontaneous chromosome doubling (PDH, number of DH plants/total green plants analyzed) (See Material and Methods section).

	PEMB *	EMB *	GP *	PGP (%)	PDH (%)	GPDH *
**(A) CLD + 2hTSA**						
CLD	67.8 a^†^	14.5 a	2.1 a	19.7 a	66.7 a	1.4 a
C+0T	82.9 a	12.2 a	1.7 a	32.4 a	69.9 a	1.2 a
C+0.1T	88.6 a	15.8 a	1.1 a	22.0 a	70.2 a	0.8 a
C+0.2T	66.5 a	12.4 a	1.2 a	12.5 a	83.3 a	1.0 a
C+0.4T	78.7 a	15.2 a	2.2 a	13.1 a	79.3 a	1.8 a
**(B) MAN + 2hTSA**						
MAN	273.7 a	33.8 a	11.9 a	70.0 a	22.8 ab	2.7 ab
M+0T	226.4 a	26.4 a	7.9 a	61.3 a	17.3 b	1.4 b
M+0.1T	262.1 a	32.6 a	12.5 a	67.2 a	34.1 a	4.3 a
M+0.2T	274.2 a	30.8 a	8.6 a	66.4 a	28.0 ab	2.4 ab
M+0.4T	254.7 a	30.9 a	8.3 a	51.3 a	12.5 b	1.0 b
**(C) CLDTSA**						
CLD	183.0 b	46.7 b	1.6 b	12.0 a	81.6 ab	1.3 b
C0T	198.8 b	47.9 b	1.2 b	9.4 a	76.8 ab	0.9 b
C0.1T	222.7 ab	58.3 ab	3.2 ab	17.0 a	86.8 a	2.7 a
C0.2T	236.1 a	64.2 a	2.6 ab	15.0 a	67.2 ab	1.7 ab
C0.4T	313.5 a	79.8 a	5.2 a	20.3 a	63.5 b	3.3 a
**(D) MANTSA**						
MAN	192.4 c	48.6 c	18.3 b	65.2 a	32.1 b	5.9 c
M0T	215.5 c	66.4 bc	26.9 ab	66.7 a	54.5 a	14.7 b
M0.1T	435.8 b	100.5 ab	33.1 ab	69.8 a	57.1 a	18.9 ab
M0.2T	563.3 ab	103.5 a	39.9 a	66.0 a	50.3 a	20.1 ab
M0.4T	646.4 a	111.8 a	40.0 a	70.4 a	62.4 a	24.9 a
**(E) TSA**						
MAN	257.1 a	50.5 a	9.4 a	63.9 a	31.2 b	2.9 b
0T	171.7 a	38.1 a	5.4 b	32.1 c	65.6 a	3.5 b
0.1T	185.3 a	44.3 a	9.1 a	46.0 ab	74.6 a	6.8 a
0.2T	170.9 a	40.0 a	7.2 ab	38.2 bc	85.3 a	6.1 a
0.4T	196.7 a	40.1 a	9.7 a	35.1 c	76.2 a	7.4 a

* Values based on 100 anthers. ^†^ Values followed by the same letter within each experiment and variable are not significantly different (*p* ˂ 0.05), according to a Duncan or a Chi square test.

**Table 2:** Microspore embryogenesis of bread wheat cvs. Pavon and Caramba after the application of 0, 0.1, 0.2, and 0.4 µM TSA simultaneously with mannitol stress treatment for 5 days at 25 °C (M0T, M0.1T, M0.2T, and M0.4T) and MAN (control culture, 0.7M mannitol for 5 days at 25 °C). Table shows TSA treatments means after statistical analysis of number of proembryos (PEMB), embryos (EMB), green plants (GP), and green DH plants (GPDH), all per 100 anthers, and percentages of green plants (PGP, number of green plants/total plants) and spontaneous chromosome doubling (PDH, percentages of DH plants/total green plants analyzed).

Cultivar	PEMB *	EMB *	GP *	PGP (%)	PDH (%)	GPDH *
***Pavon***						
MAN	211.7 b^†^	59.2 b	25.9 b	73.7 a	28.0 b	7.2 b
M0T	185.8 b	70.7 ab	33.1 ab	66.3 a	52.7 a	17.8 ab
M0.1T	402.9 ab	113.4 a	43.8 ab	75.9 a	56.2 a	24.6 ab
M0.2T	563.0 a	115.8 a	52.3 ab	75.5 a	44.0 ab	23.0 ab
M0.4T	516.1 a	120.3 a	52.1 a	71.0 a	58.9 a	30.7 a
***Caramba***						
MAN	162.2 c	30.4 c	5.1 b	49.4 a	53.2 b	2.7 b
M0T	287.1 bc	55.7 bc	12.0 ab	67.7 a	56.5 b	6.8 ab
M0.1T	499.5 ab	78.9 ab	13.5 ab	59.4 a	60.0 b	8.1 ab
M0.2T	564.0 ab	74.9 ab	11.4 ab	45.0 a	79.3 a	9.0 ab
M0.4T	890.3 a	97.7 a	18.2 a	69.5 a	72.6 ab	13.2 a

* Values based on 100 anthers. ^†^ Values followed by the same letter within each cultivar and variable are not significantly different (*p* ˂ 0.05), according to a Duncan or a Chi square test.

## Supplementary Materials

The following are available online at https://www.mdpi.com/2223-7747/9/11/1442/s1, Figure S1: Scheme of the different strategies used to induce microspore embryogenesis in wheat anther culture by the application of trichostatin A (TSA) in combination with or without different stress treatments.

## Abbreviations

BLAST	Basic local alignment search tool
CLD	Control cold treatment (7–9 days at 4 °C)
MAN	Control mannitol treatment (0.7 M mannitol at 25 °C for 5 days)
C0T	Cold treatment with 1% DMSO
C0.1T	Cold treatment with 0.1 µM TSA
C0.2T	Cold treatment with 0.2 µM TSA
C0.4T	Cold treatment with 0.4 µM TSA
C + 0T	Cold treatment plus 2 h with 1% DMSO
C + 0.1T	Cold treatment plus 2 h with 0.1 µM TSA
C + 0.2T	Cold treatment plus 2 h with 0.2 µM TSA
C + 0.4T	Cold treatment plus 2 h with 0.4 µM TSA
DAPI	4′,6-Diamidine-2’-phenylindole dihydrochloridl
DH	Doubled haploid
EMB	Number of embryos/100 anthers
FM	Fresh microspores
GP	Number of green plants/100 anthers
GPDH	Number of green DH plants/100 anthers
ME	Microspore embryogenesis
M0T	Mannitol treatment with 1% DMSO
M0.1T	Mannitol treatment with 0.1 µM TSA
M0.2T	Mannitol treatment with 0.2 µM TSA
M0.4T	Mannitol treatment with 0.4 µM TSA
M + 0T	Mannitol treatment plus 2 h with 1% DMSO
M + 0.1T	Mannitol treatment plus 2 h with 0.1 µM TSA
M + 0.2T	Mannitol treatment plus 2 h with 0.2 µM TSA
M + 0.4T	Mannitol treatment plus 2 h with 0.4 µM TSA
PDH	Percentage of DH plants/total green plants analyzed
PEMB	Number of proembryos /100 anthers
PGP	Number of green plants/total plants
RNA	Ribonucleic acid
RT	Room temperature
SLM	“Star-like” morphology
TSA	Trichostatin A
0T	1% DMSO treatment for 2 h
0.1T	0.1 µM TSA treatment for 2 h
0.2T	0.2 µM TSA treatment for 2 h
0.4T	0.4 µM TSA treatment for 2 h
