# Supplementary material for: Trichostatin A Affects Developmental Reprogramming of Bread Wheat Microspores towards an Embryogenic Route
Source: Plants (Basel). 2020 Oct 26;9(11):1442. doi: 10.3390/plants9111442 (PMC7693754; doi:10.3390/plants9111442)
Supplement: Supplementary file 1 [file plants-09-01442-s001.pdf]

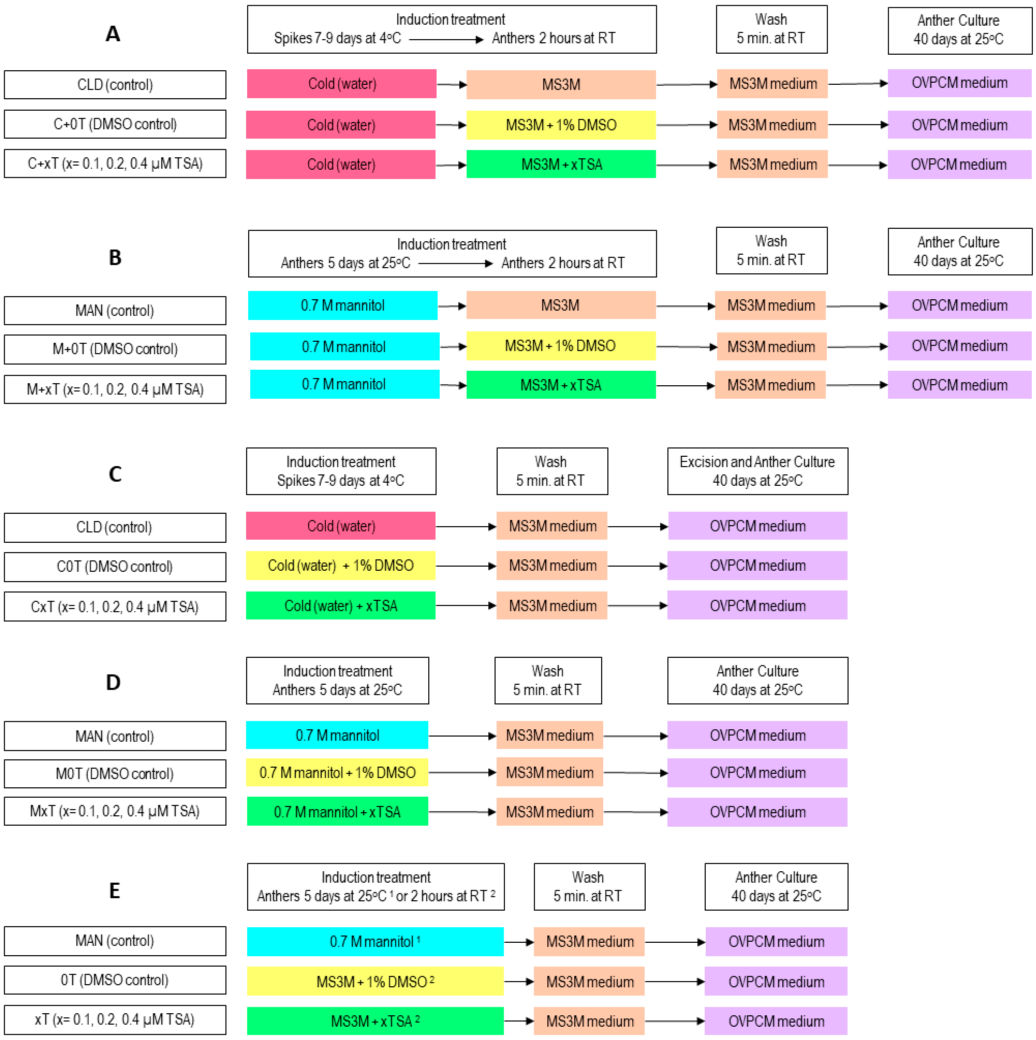

Supplementary Figure 1. Scheme of the diverse strategies used to induce microspore embryogenesis in wheat anther culture by the application of trichostatin A (TSA) in combination with or without different stress treatments. Concentrations of 0.1, 0.2, and 0.4 μM TSA dissolved in 1% DMSO (0.1T, 0.2T, and 0.4T), and control cultures with 0% DMSO (0T) were tested in all strategies. Anthers cultured with 0.7 M mannitol for 5 days at 25 °C or spikes at 4 °C for 7–9 days were used as control experiments depending on the strategy used. (**A**) Application of TSA after cold stress treatment, **(B)** application of TSA after mannitol stress treatment, **C**) application of TSA in combination with cold stress treatment, (**D**) application of TSA in combination with mannitol stress treatment, and (**E**) application of TSA as a unique anther treatment. RT = 25 °C.
